# An Effective Approach to Improving Low-Cost GPS Positioning Accuracy in Real-Time Navigation

**DOI:** 10.1155/2014/671494

**Published:** 2014-06-25

**Authors:** Md. Rashedul Islam, Jong-Myon Kim

**Affiliations:** Department of Electrical, Electronics and Computer Engineering, University of Ulsan, Building No. 7, Room No. 308, 93 Daehak-ro, Nam-gu, Ulsan 680-749, Republic of Korea

## Abstract

Positioning accuracy is a challenging issue for location-based applications using a low-cost global positioning system (GPS). This paper presents an effective approach to improving the positioning accuracy of a low-cost GPS receiver for real-time navigation. The proposed method precisely estimates position by combining vehicle movement direction, velocity averaging, and distance between waypoints using coordinate data (latitude, longitude, time, and velocity) of the GPS receiver. The previously estimated precious reference point, coordinate translation, and invalid data check also improve accuracy. In order to evaluate the performance of the proposed method, we conducted an experiment using a GARMIN GPS 19xHVS receiver attached to a car and used Google Maps to plot the processed data. The proposed method achieved improvement of 4–10 meters in several experiments. In addition, we compared the proposed approach with two other state-of-the-art methods: recursive averaging and ARMA interpolation. The experimental results show that the proposed approach outperforms other state-of-the-art methods in terms of positioning accuracy.

## 1. Introduction

The global positioning system (GPS) is a satellite-based positioning system that was implemented and experienced rapid growth in the last two decades [1]. Location-based services (LBSs) refer to services that function based on the location information and context of users. Location-based mobile applications leverage GPS technology embedded in devices to determine the current user location [2, 3]. There are several different technologies for providing different ranges of location-based services using mobile communication devices, including Wi-Fi finger printing, wireless sensor networking, and WLAN for indoor positioning, as well as a small range of outdoor positions [4–8]. However, GPS has become the best and foremost outdoor positioning system for the wide range of location-based services [9]. GPS-based LBSs are widely used in applications such as tourist information, finding the nearest point of interest (POI) [10], military data sharing with location and user validation [11], aircraft monitoring, vehicle tracking, vehicle-to-vehicle communication, vehicle-to-infrastructure communication, and vehicle load distribution on roads [12–14].

**Figure 1:**
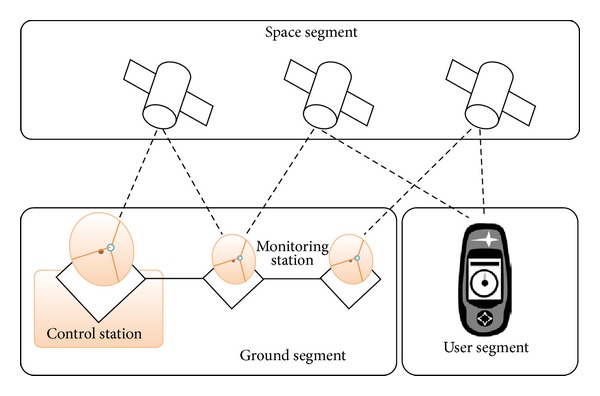
Segments of a GPS system [22].

GPS can measure the time, altitude, longitude, and latitude using available satellite signals. The idea of satellite positioning was developed in the early 1960s [1], but GPS was not deployed until the 1970s and then only by the US Department of Defense (DoD) for military purposes (positioning, navigation, and weapons aiming). In fact, the DoD included a distortion in the GPS signal called selective availability (SA) so that other people could not use GPS with high precision. Practical operation of GPS started in 1978; not until 1995 was GPS a fully operational positioning system. On May 1, 2000, selective availability was deactivated [15]. GPS is currently used worldwide for civilian applications (e.g., driving assistance, topography, and atmosphere study). GPS architecture comprises three spatial, control, and user segments, as depicted in Figure 1. The spatial segment includes 24 satellites in orbit more than 20,000 km from the Earth, six orbital levels, and a 12-hour period. The control segment includes Earth stations that control the satellites' trajectories. Finally, the users segment includes GPS receivers [16]. Typically, four or more satellites are needed to accurately calculate a position [15].

GPS determines the position of a target by measuring the propagation delay of signals from the satellites to the GPS receiver. The GPS operation principle is based on measurement of the range of distances between the receiver and satellites [17, 18] using propagation delay and speed of light. The basic formula is described by

(1) $$\begin{array}{c} \text{Dist}=\text{Dela}{\text{y}}_{\text{propagation}}\times \text{Spee}{\text{d}}_{\text{light}}. \end{array}$$

**Figure 2:**
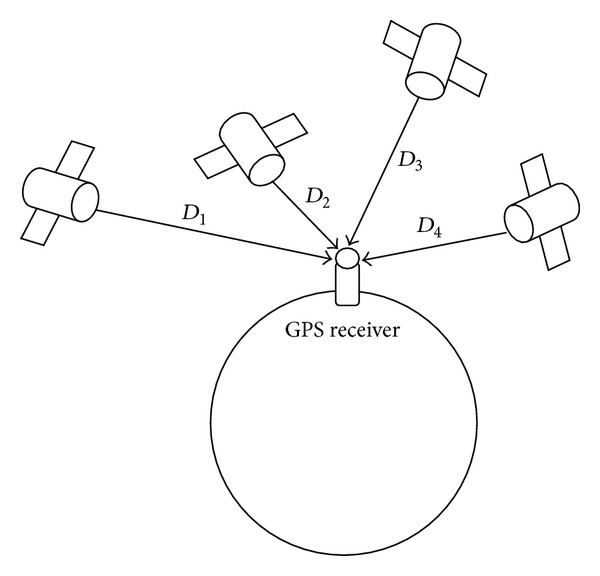
Basic orientation of satellites and a GPS receiver.

The positioning value of a GPS receiver is calculated from the signal received from satellites.  Figure 2 shows the basic orientation of satellites and a GPS receiver. In addition, positioning values are calculated by

(2) D=C(T−t+tc)=(X−x)2+(Y−y)2+(Z−z)2,

where *C* is speed of light, *T* is the time at which GPS satellites transmitted their signals (these times are provided to the receiver as part of the transmitted information), *t* is the time at which the signals from GPS satellites are received, and *X*, *Y*, and *Z* are coordinates of the GPS satellite. The receiver solves these equations simultaneously to determine *x*, *y*, *z*, and *t*_*c*_, where *x*, *y*, and *z* are the receiver's latitude, longitude, and altitude values, respectively, and *t*_*c*_ is the time correction of the GPS receiver's clock.

In GPS, mobility, reliability, and accuracy are still challenging issues [19]. In general, the error in longitude and latitude coordinates is 10–15 meters in 95% of readings [20]. In addition, position accuracy can be reduced by different error sources such as satellite geometry, multipath effect, atmospheric effects, clock inaccuracies, rounding errors, and receiver noise [21]. In practical applications, the positioning algorithms utilize the data received by the GPS receiver, which suffers from positioning error and fluctuations from the actual path.

This paper proposes an efficient location-determination approach based on direction and velocity averaging, which estimates the precious position using the latitude, longitude, and velocity values without calculating a large number of the previous databases which include coordinate position values of the past timestamp location. In addition, the proposed approach addresses different error sensitivity issues.

The rest of this paper is organized as follows.  Section 2 presents the background research.  Section 3 describes the proposed method.  Section 4 presents experimental results and compares the performance of the proposed method and other conventional methods. Finally, Section 5 concludes the paper.

## 2. Related Work

Many researchers have worked to improve the positioning accuracy of GPS using different methods. The existing methods can be classified into three categories [9]: (1) the use of expensive modules such as WAAS, DGPS, and AGPS; (2) the use of an additional auxiliary peripheral module to supplement the GPS computation; and (3) the implementation of software algorithms for more accurate computation of GPS data, such as the Kalman filter and Wiener filter [15, 17]. The Kalman filter is a recursive estimator that uses the previous state to probabilistically estimate current position and velocity. In other words, it uses the previous state to predict the position of the next phase and assumes that the previous state is correct. However, this method has no capability for self-correction [9]. For error reduction of a low-cost GPS receiver, Refan and Palangi proposed the recursive averaging and autoregressive-moving average (ARMA) interpolation methods, and ARMA modeling showed satisfactory performance [1]. Huang and Tsai proposed perceptive GPS (PGPS), which consists of two phases: (1) a training phase where the system adopts a learning algorithm of the hidden Markov model (HMM) in order to perceive the carrier's behavior and (2) a rectification phase that uses data from the training phase to convert the carrier's behavior to position data in real time [9].

## 3. The Proposed Method

**Figure 3:**
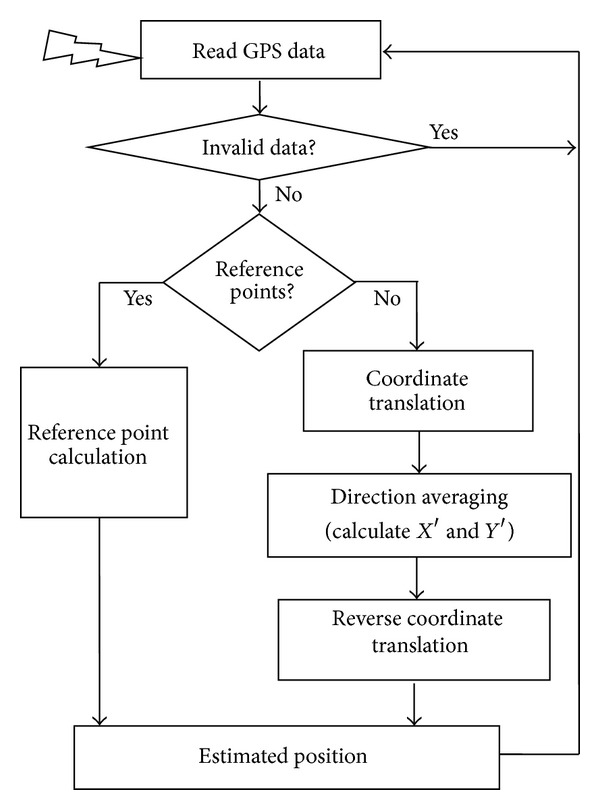
A basic flow diagram of the proposed method.

In contrast to previous studies that did not address the data fluctuation in movement direction and velocity, the proposed method reduces positioning errors by employing movement averaging, velocity, and the distance among waypoints. To improve positioning accuracy, the proposed method focuses on the following three issues: (a) determining a more accurate reference point, (b) reducing rounding errors using a coordinate translation process, and (c) estimating direction averaging and speed averaging of waypoints. Determination of an accurate reference point provides more accurate calculation in the subsequent steps. The coordinate translation before and after directional averaging to calculate latitude (*X*′) and longitude (*Y*′) reduces rounding errors. Finally, the proposed method calculates *X*′ and *Y*′ of a new coordinate position corresponding to the local coordinate.  Figure 3 shows a basic flow diagram of the proposed method.

### 3.1. Invalid Data Check

First, the invalid data check module in Figure 3 filters the invalid GPS data from the receiver by checking the valid data flag, the number of connected satellites, latitude, and longitude values.

### 3.2. Obtaining an Accurate Reference Point

The GPS data from the receiver can fluctuate at every timestamp. An accurate starting position is needed and will be the reference point for further estimations. To obtain the reference point, we use long-term averaging, which is performed on the fixed position data in order to determine the reference position of that receiver [1]. For long-term averaging, the position and time data of a fixed position are collected over a long duration, and the long-term average values of both latitude and longitude are calculated using (3). This long-term averaging value is used as a reference point in subsequent steps:

(3) $$\begin{array}{c} {\text{AVG}}_{x}=\frac{1}{N}\overset{N}{\underset{i=1}{\sum }}x_{i}\;\;{\text{AVG}}_{y}=\frac{1}{N}\overset{N}{\underset{i=1}{\sum }}y_{i}, \end{array}$$

where *x* is the latitude, *y* is the longitude, and *N* is the number of timestamps.

### 3.3. Coordinate Translation

Coordinate translation relocates the coordinate center position to any location other than the original center [0,0], and coordinate positions are measured by the new coordinate center. In general, a point in global positioning is represented by three coordinate values including latitude (N or S), longitude (E or W), and altitude, and the coordinate value is presented in degrees, minutes, and seconds. The main motivation of coordinate translation is to reduce rounding errors for more precise position determination. The data calculation in GPS generally uses values in decimal form converted from real data, which induces rounding error. This rounding results in loss of some figures after the decimal point; this loss most greatly impacts the seconds measurement of latitude or longitude during new point estimation. This error can be reduced by using the coordinate translation.

**Figure 4:**
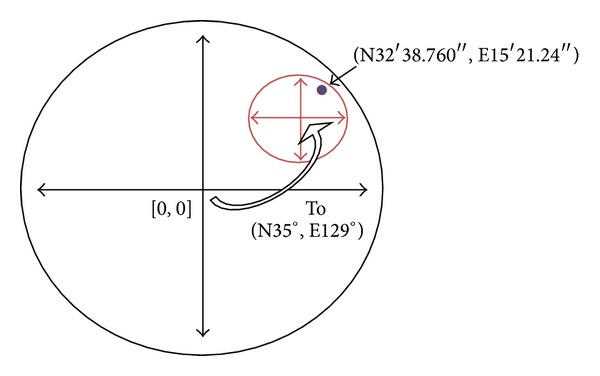
Coordinate translation between global coordinates and local coordinates.

The coordinate value is presented in degrees, minutes, and seconds based on the global coordinate center [0, 0]. Coordinate translation relocates the coordinate center from [0, 0] to any required local coordinate center. Therefore, all positions in the current navigation area are calculated relative to the local coordinate center. For example, assume that a position (P) on Earth has the coordinate value [N35° 32′ 38.760′′, E129° 15′ 21.24′′]. After translating (relocating) the coordinate center [0, 0] to the local point [N35°, E129°], the coordinate value of the position (P) will be [N32′ 38.760′′, E15′ 21.24′′] from that local coordinate center.  Box 1 shows sample data collected from a GPS receiver, and Figure 4 shows the coordinate translation between global coordinates and local coordinates.

### 3.4. Direction Averaging for Calculating *X*′ and *Y*′

**Figure 5:**
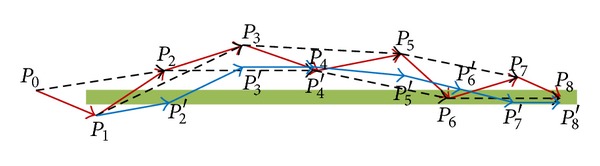
An example for calculating position using the proposed model.

This section describes the proposed direction averaging model used to estimate new coordinate values in local coordinates. The proposed model calculates new coordinate values from the translated coordinate data collected from a GPS receiver. This calculation of the directions uses the speed and distance of past and present steps. At every step, the coordinate values of the present and last two waypoints and the velocities between those waypoints are determined. Those values are used to calculate the direction angles from one point to the next and the distances between those steps. The calculated direction angle values and distance values are used to calculate a new position in every iteration.  Figure 5 shows a sample model for calculating position using the proposed model. The points *P*_0_, *P*_1_,…, *P*_8_ are original waypoints, and the points *V*_0_, *V*_1_,…, *V*_8_ are velocities. The system calculates the direction angle and distance from *P*_0_ to *P*_1_ and from *P*_1_ to *P*_2_ and then calculates the new waypoint *P*_2_′ by combining direction and averaged distances, where the averaged distances are calculated using the actual distance and velocity of the previous points. In this model, the first point, starting point *P*_0_, is a reference point calculated from long-term averaging. This execution continues until the new waypoints *P*_2_′, *P*_3_′,…, *P*_8_′ are estimated.

The red line in Figure 5 is the original path, the blue line is the estimated path, and the dashed line indicates a combined direction of two angles for three corresponding points. Equations (4) represent the generalized formula for the estimation process of position values (*X*′ and *Y*′).

Consider the following:

(4) $$\begin{array}{c} X_{n+1}^{\prime }=X_{n}^{\prime }+D\times \cos\left(\text{ta}{\text{n}}^{-1}\frac{y_{n+1}-y_{n-1}}{x_{n+1}-x_{n-1}}\right),\\ Y_{n+1}^{\prime }=Y_{n}^{\prime }+D\times \sin\left(\text{ta}{\text{n}}^{-1}\frac{y_{n+1}-y_{n-1}}{x_{n+1}-x_{n-1}}\right), \end{array}$$

where

(5) $$\begin{array}{c} D=\sqrt{{\left(x_{n+1}-x_{n}\right)}^{2}+{\left(y_{n+1}-y_{n}\right)}^{2}}\times \frac{V_{n}}{V_{n-1}}. \end{array}$$

In (4), *X*′ and *Y*′ are new estimated coordinates, *x* and *y* are real translated coordinate values, *V* is the velocity of position data, and *D* is the calculated distance.

## 4. Experimental Results and Performance Evaluation

In the experiment, we used a GARMIN GPS 19xHVS receiver for data collection. We attached a receiver at the top of the car and inside the car we used a laptop with our simulation software as a data processing terminal to calculate and plot the data in Google Maps. A serial port with a serial-to-USB converter was used for communication between the GPS receiver and the laptop.  Table 1 describes the receiver specifications.

**Figure 6:**
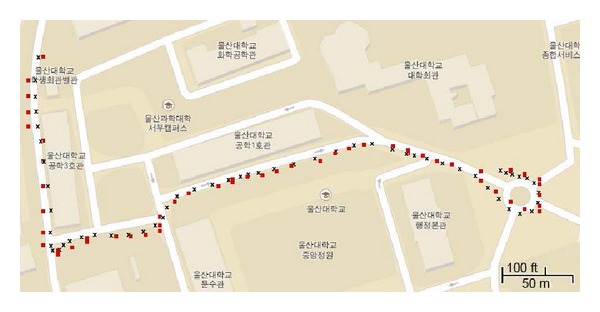
Result of the proposed method, where the red blocks are real GPS data received from the receiver and the black crosses are the results of the proposed method.

We collected data from a fixed location as well as while driving. We performed the experiment several times in different locations.  Figure 6 shows the experimental results using the proposed method, where the red blocks represent the real data received from the GPS receiver and the black crosses represent the data processed using the proposed method.

**Figure 7:**
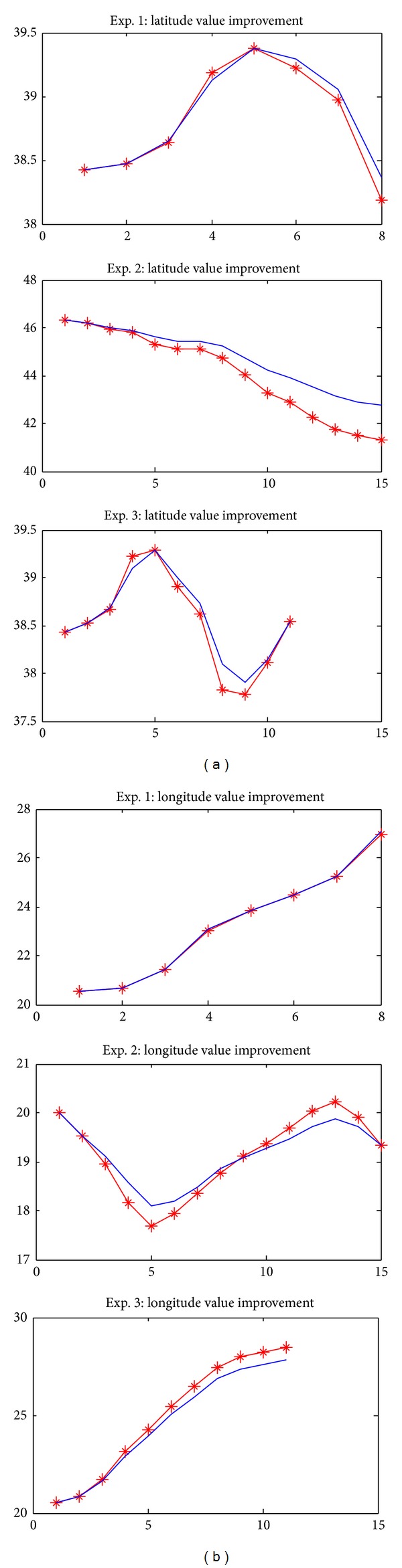
Coordinate position value improvements: (a) latitude and (b) longitude (*x*-axis is data points and *y*-axis is coordinate value in seconds).

Figure 7 shows a more detailed view of the improvement in latitude and longitude values of a few points in the experiments using the proposed model. The *x*-axis represents the data points of the experiment, and the *y*-axis is the coordinate value (second component), where the red (∗) line represents the coordinate component value of real data from the GPS receiver and the blue (—) line represents the data processed using the proposed method.

**Figure 8:**
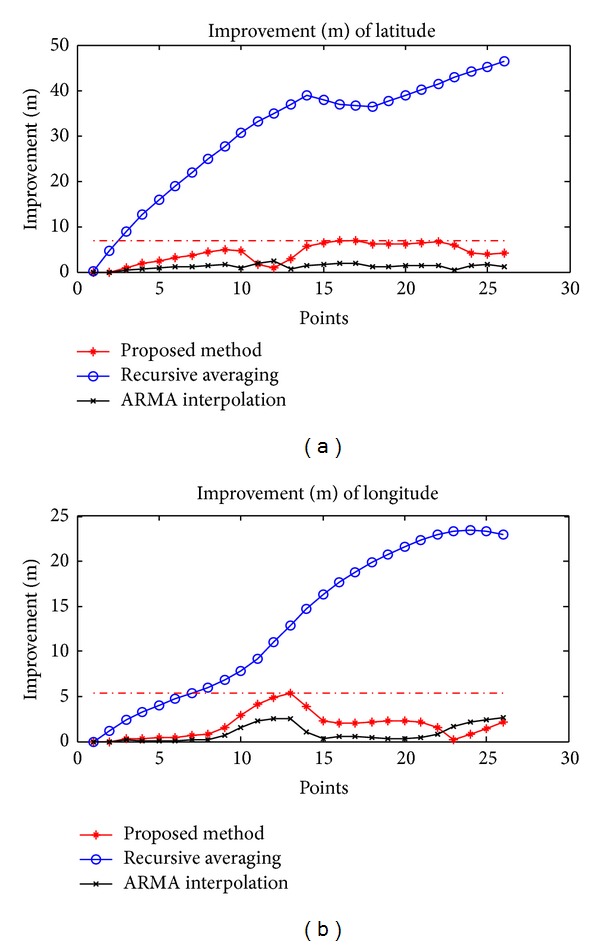
Improvement of the proposed method, recursive averaging, and ARMA interpolation: (a) latitude values and (b) longitude values.

As mentioned previously, a low-cost GPS receiver has an error of 10–15 meters (a combination of different sources of error) in 95% of the readings [20]. From the error analysis of the proposed method, we obtain an improvement of 4–10 meters while driving. We also compare the proposed method with existing recursive averaging and ARMA interpolation methods [1].  Figure 8 shows the improvement (in meters) of the proposed method and the recursive averaging and ARMA interpolation methods.

We perform experiments several times in different places.  Table 2 summarizes the results of six experiments. In several different experiments, we observe that the recursive averaging method shows much improvement in positioning accuracy, but estimated waypoints are out of road because recursive averaging of latitude and longitude makes more errors in the way of curve. On the other hand, the ARMA interpolation has two coefficient parameters which affect the accuracy of new estimated points. Overall, the proposed method outperforms other methods in terms of positioning.

## 5. Conclusions

This paper presented a new, effective method for improving GPS positioning accuracy for low-cost standard GPS navigation. The proposed method estimates positioning accuracy based on direction angle, velocity, and distance using an accurate reference point and long-term averaging, rounding error reduction via coordinate translation, and an invalid data check. Using a GARMIN GPS 19xHVS receiver, we compared the performance of the proposed method and the other existing approaches. Experimental results showed that the proposed method provides 4–10-meter improvement and outperforms other approaches in positioning accuracy by reducing significant data fluctuation of real data from low-cost GPS receivers.

## Figures and Tables

**Box 1:**
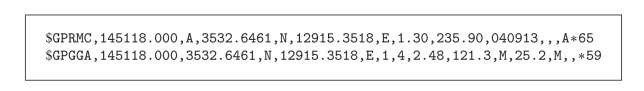
Collected sample data from a GPS receiver.

**Table 1:** The receiver specifications.

Type	Updated rate	Accuracy	Provided data
Standard GPS receiver	1, 5, and 10 records per second	Less than 15 meters with 95% typical	Pseudorange, integrated carrier phase, Doppler shift, satellite ephemeris, and processed data

**Table 2:** Performance of the proposed method and the two state-of-the-art methods.

Exp. number	Proposed method		Recursive averaging		ARMA interpolation	
	Latitude	Longitude	Latitude	Longitude	Latitude	Longitude
1	7.3393	7.2127	90.6028	48.3929	5.1617	6.2125
2	7.9177	5.2747	87.9283	59.9363	3.4236	3.5771
3	6.9827	4.8645	85.7571	76.5026	7.7118	4.5644
4	5.9856	9.7594	39.0915	57.3822	2.4497	4.9815
5	6.3945	6.1221	23.5941	35.9982	1.4216	4.2024
6	6.8250	5.3022	56.2654	23.4819	2.4113	2.6461
